# CRISPR/Cas9-Mediated Mutagenesis of *OsERF94* Enhances Pre-Harvest Sprouting in Rice

**DOI:** 10.1186/s12284-026-00923-7

**Published:** 2026-06-27

**Authors:** Man Bo Lee, Ha Neul Lee, Sang-Ho Chu, Yong-Jin Park, Chang-Wook Park, In-Jung Lee, Lae-Hyeon Cho, Jae Yoon Kim

**Affiliations:** 1 Department of Plant Resources, College of Industrial Science, Kongju National University, Yesan, 32439 South Korea; 2 Department of Applied Biosciences, Kyungpook National University, Daegu, 41566 South Korea; 3 Department of Plant Bioscience, College of Natural Resources and Life Science, Pusan National University, Miryang, 50463 South Korea

**Keywords:** *OsERF94*, CRISPR/Cas9, Pre-harvest sprouting, Germination, GA biosynthesis, GA deactivation

## Abstract

Pre-harvest sprouting (PHS), where seeds germinate on panicles before harvest under humid conditions, is a serious global issue in cereal crop production, including rice. Fine-mapping of the previously reported chromosome 4 locus identified *OsERF94* as a strong candidate gene for functional validation. In this study, we investigated the role of *OsERF94* in PHS using CRISPR/Cas9 gene editing. The CRISPR/Cas9-mediated mutagenesis of *OsERF94* induced frameshift mutations, resulting in a loss-of-function of *OsERF94* in the 1-I-ET and 2-D-ET lines. The 1-I-ET and 2-D-ET lines exhibited significantly higher germination rates under PHS conditions compared to the wild type, indicating increased susceptibility to PHS. Whole-genome re-sequencing confirmed that few or no mutations could be detected at off-target candidate sites in both edited lines, ensuring the precision of the CRISPR/Cas9 gene editing. A transcriptome analysis revealed altered expression patterns of several GA-related genes, including *OsLOL1*, *OsKO3*, *OsGA3ox2*, and *OsGA2ox5* in the *OsERF94* mutant lines. The up-regulation of GA biosynthetic genes and the down-regulation of GA deactivation genes observed in both the *OsERF94* mutant lines suggest possible alterations in GA metabolism during the early stages of PHS. Transient luciferase reporter assays using a single-luciferase system suggested that *OsERF94* may be associated with changes in the promoter activities of several GA- and ethylene-related genes. These findings suggest that *OsERF94* may contribute to the regulation of PHS, potentially through moderation of GA- and ethylene-related pathways. Overall, this study improves our understanding of the molecular role of *OsERF94* in PHS and highlights its potential as a target for the genetic improvement of PHS resistance in rice-breeding programs.

**Supplementary Information:**

The online version contains supplementary material available at https://doi.org/10.1186/s12284-026-00923-7.

## Introduction

Pre-harvest sprouting (PHS) refers to a phenomenon where seeds germinate on the panicles before harvest under continuously rainy and humid weather (Li et al. 2004). PHS has become a serious global issue during the agricultural production of cereal crops such as rice, with the average annual financial loss due to PHS has been estimated to be one billion dollars worldwide (Tai et al. 2021; Hull et al. 2024). PHS also affects grain quality by enhancing the activity of oxidoreductases and hydrolases in cereal grains, resulting in the loss of nutrients such as starch, protein, and oil (Li et al. 2004). The eating and cooking quality of rice grains were lower in rice plants affected by PHS, as were the amylose and total starch contents (Zhang et al. 2020a, b). However, cultivated crops generally exhibit lower dormancy levels compared to their wild ancestors, which makes modern crop varieties more susceptible to PHS (Tai et al. 2021). Due to global climate change, heavy rainfall in specific regions can lead to more frequent occurrences of PHS (Hu et al. 2025). Therefore, developing PHS-resistant varieties has become a key objective in rice-breeding programs, alongside the discovery of PHS-resistant genes (Lee et al. 2021).

PHS occurs primarily due to an imbalanced dormancy/germination ratio (Tai et al. 2021). Seed dormancy and germination are distinct biochemical and physiological processes regulated by various internal factors, such as plant hormones, carbohydrate metabolites, and reactive oxygen species (Hilhorst and Karssen 1992; Yu et al. 2016; Omoarelojie et al. 2022). Among plant hormones, abscisic acid (ABA) and gibberellin (GA) play critical roles in antagonistically controlling dormancy and germination (Yaish et al. 2010; Sohn et al. 2021). ABA promotes dormancy formation and maintenance, whereas GA induces germination by counteracting ABA effects on dormancy (Finkelstein et al. 2008). In rice, *OsVP1* activates *Sdr4*, a key regulator of pre-harvest sprouting, by binding to its promoter (Chen et al. 2021). *OsVP1* and *Sdr4* play roles in the biosynthesis and signaling pathways of ABA and GA, regulating seed dormancy. A rice mutant with delayed germination and semi-dwarfism was identified, caused by mutations in *OsKO1* (Zhang et al. 2020a, b). The mutation inhibits GA biosynthesis, impairing starch mobilization and reducing ABA signaling, ultimately delaying seed germination.

Ethylene also plays important roles in the regulation of dormancy and germination (Corbineau et al. 2014). The *Arabidopsis reduced dormancy 3* mutant is caused by a loss-of-function mutant of the ethylene receptor ETHYLENE RESPONSE1 (ETR1) (Li et al. 2019). ETR1 regulates seed dormancy and germination partially through the DELAY OF GERMINATION1 pathway. Ethylene counteracts the inhibitory effects of ABA on germination by modulating ABA metabolism and signaling pathways (Linkies and Leubner-Metzger 2011). Additionally, ethylene and GA work synergistically to promote seed germination (Corbineau et al. 2014). Ethylene response factors (ERFs), a family of APETALA2/ETHYLENE RESPONSE FACTORS (AP2/ERFs), regulate genes involved in hormone and stress signaling (Müller and Munné-Bosch 2015; Phukan et al. 2017).

In rice, CRISPR/Cas9 gene editing has been applied for gene-function analyses and for the development of stress-resistant varieties, including those resistant to PHS (Romero and Gatica-Arias 2019; Kim et al. 2024). The CRISPR/Cas9 gene editing of *OsABA8ox* resulted in frameshift mutations in gene-edited rice lines (Fu et al. 2022). Loss-of-function in *OsABA8ox* enhances seed dormancy by elevating endogenous ABA levels and modulating ABA signaling. Overexpression and CRISPR/Cas9 gene editing of *OsMFT2*, which encodes a phosphatidylethanolamine-binding protein, resulted in different responses to PHS (Zhang et al. 2025). Overexpression lines exhibited strong seed dormancy and weak PHS, whereas gene-edited lines showed strong PHS.

**Fig. 1:**
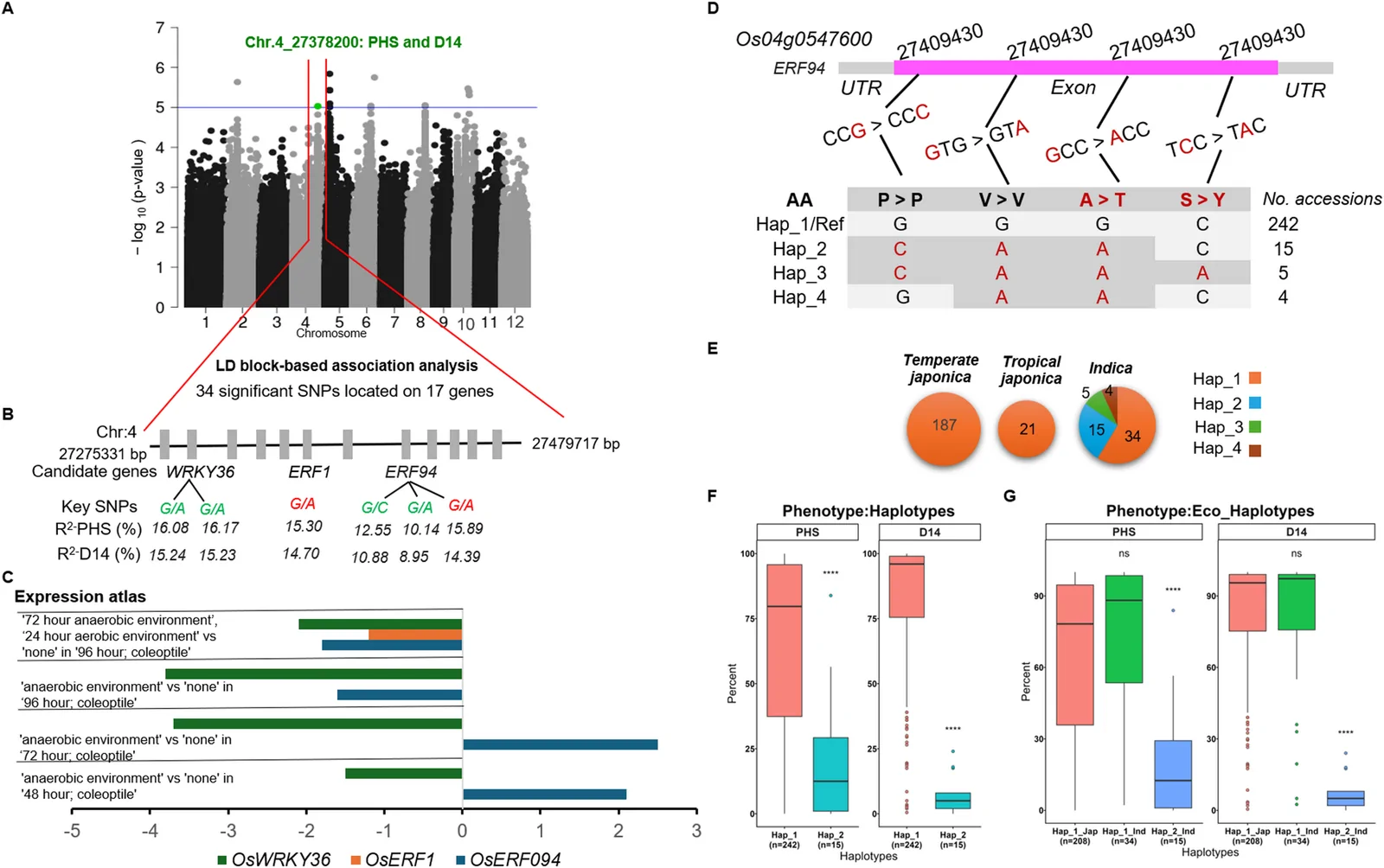
Fine mapping of the Chr4 LD block identifies *OsERF94* as a strong candidate gene underlying PHS and detached-grain germination at 14 days (D14) in cultivated rice. **A** Manhattan plot highlighting the dual-trait lead SNP, Chr4_Pos27378200 (Min et al. 2024). **B** LD block surrounding Chr4_Pos27378200 on chromosome 4, showing the physical positions of candidate genes, including *OsWRKY36* (*Os04g0545000*), *OsERF1* (*Os04g0546800*), and *OsERF94* (*Os04g0547600*). **C** Expression Atlas-based expression profiles of *OsWRKY36*, *OsERF1*, and *OsERF94* under germination-related anaerobic/low-oxygen conditions. **D** Gene structure and coding haplotypes of *OsERF94* identified in 280 cultivated rice accessions **E** Distribution of *OsERF94* haplotypes across ecotype groups. **F** Haplotype–phenotype associations for PHS and D14. **G** Ecotype-based comparison of phenotypes by haplotype. Boxplot comparisons are based on estimated marginal means, with significance thresholds: ns = non-significant, * *p* < 0.05, ** *p* < 0.01, and **** *p* < 0.0001

In our previous GWAS on a cultivated rice panel, a compressed mixed linear model identified 15 and 76 significant SNPs for PHS and detached-grain germination (D14), respectively (Min et al. 2024). Subsequent LD-block–based association analysis using a general linear model within a ± 100 kb window of the dual-trait lead SNP Chr4_Pos27378200 identified 34 coding SNPs across 17 genes (Fig. 1A), including *OsERF1* (*Os04g0546800*), *OsWRKY36* (*Os04g0545000*), and *OsERF94* (*Os04g0547600*). Within this hub, three SNPs within *OsERF94* explained 8.95–15.89% of phenotypic variation in PHS and D14 (Fig. 1B). *OsERF1* was recently edited via CRISPR-Cas9/HDR with a geminiviral replicon and the engineered ERF1-hdr line (carrying a 1 bp SNP and a 6 bp insertion) exhibited enhanced seed dormancy and PHS resistance (Kim et al. 2024). Consistently, ABA-pathway genes, such as *PYL*, *SnRK2*, *ABI3*, *ABI5*, and *Sdr4*, were up-regulated, whereas ethylene/GA markers (*EIN3*, *GID1*, *GYMYB*) were down-regulated in the ERF1-hdr line compared to the wild type (WT) (Kim et al. 2024). In parallel, *OsWRKY36* functions as a GA-signaling repressor, stabilizing *SLR1* (a DELLA-like inhibitor); overexpression produces a semi-dwarf/small-grain phenotype (sgsd3), while knock-down and knock-out increases grain size (Lan et al. 2020). These findings suggest that the Chr4_Pos27378200 region may represent a regulatory hub where transcription factors associated with ABA, ethylene, and GA pathways are located and may contribute to the regulation of seed dormancy.

Given its strong genetic signal, membership in the AP2/ERF family, and positional convergence within this hormone hub, we hypothesized that *OsERF94* may be involved in the regulation of seed dormancy and PHS. We therefore investigated its role using gene editing and transcriptomic analyses.

## Results

### *OsERF94*, a Candidate Gene Associated with Pre-Harvest Sprouting in Rice

Fine-mapping around maker Chr4_Pos27378200 (associated with both PHS and D14) delineated an LD block enriched for transcription factors and containing *OsWRKY36*, *OsERF1*, and *OsERF94* (Fig. 1A and B). To prioritize candidates, we queried public expression resources for condition-specific profiles relevant to seed germination and anaerobic germination conditions associated with PHS. In the Expression Atlas database (https://www.ebi.ac.uk/gxa/home), only *OsERF94* showed up-regulation under 48- and 72-hours anaerobic germination-linked and low-oxygen/anaerobic contexts, whereas *OsERF1* displayed moderate responsiveness and *OsWRKY36* remained comparatively low in these conditions (Fig. 1C). Subsequently, we investigated haplotypes of *OsERF94* in a panel of 280 cultivated rice accessions. ‘Nipponbare’-referenced variant calling identified four coding SNPs; two synonymous and two non-synonymous within *OsERF94* (Fig. 1D), three of which were also captured as significant SNPs in the LD-block association scan (Fig. 1B and D). These variants define four haplotypes, with Hap_1 being predominant (*n* = 242) and Hap_2 the next most frequent (*n* = 15) (Fig. 1D). Ecotype based classification revealed that all *japonica* accessions grouped into Hap_1, while among 58 *indica* accessions, 34 were Hap_1 and 15 were Hap_2 (Fig. 1E). Haplotype–phenotype association tests showed a significant difference in PHS and D14 between Hap_1 and Hap_2 (Fig. 1F). When evaluated with the ecotype context, differences remained non-significant within *japonica* and *indica* subsets of Hap_1, but were significant when comparing Hap_1 (*indica*/*japonica*) against Hap_2 (*indica*) (Fig. 1G). Overall, the LD co-localization, condition-relevant expression bias, and haplotype–phenotype separation support *OsERF94* as the lead candidate underlying the Chr4 PHS signal, particularly in *indica* backgrounds. Taken together, the convergence of positional association, condition-specific expression patterns, and haplotype–phenotype relationships strongly supports the prioritization of *OsERF94* for functional validation. To validate this hypothesis in *japonica* rice, we generated CRISPR/Cas9 loss-of-function mutants of *OsERF94* in the ‘Nipponbare’ background for functional validation.

### Development of *OsERF94* Mutant Lines via CRISPR/Cas9

**Fig. 2:**
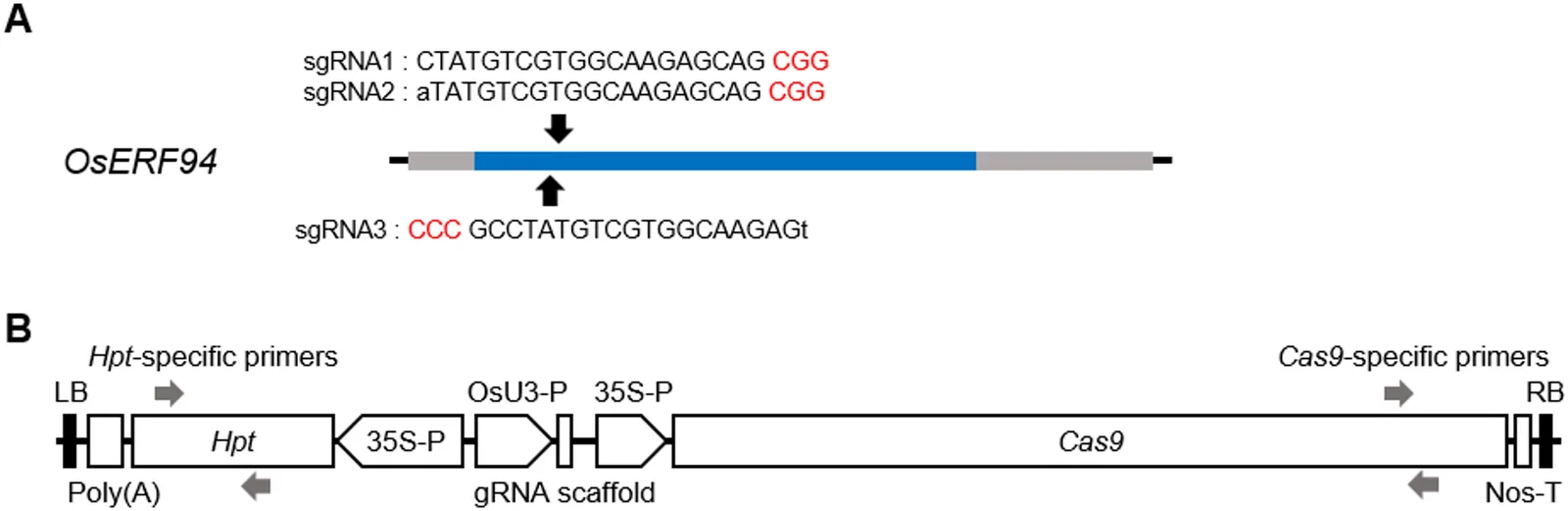
Schematic representation of *OsERF94* and the CRISPR/Cas9 vector. **A** A schematic diagram of *OsERF94* and the target sequences of each sgRNA. The blue box indicates the exon, and gray boxes indicate the untranslated regions. The black arrows indicate the locations of the target sequences of the sgRNAs. PAM sequences are shown in red. **B** A schematic diagram of the T-DNA region of the CRISPR/Cas9 vector. The gray arrows indicate the target sequences of *Hpt*-specific primers and *Cas9*-specific primers. LB, left border; Poly(A), poly(A) tails; *Hpt*, *hygromycin phosphotransferase*; 35 S-P, cauliflower mosaic virus 35 S promoter; *OsU3*-P, rice *U3* promoter; *Nos*-T, *nopaline synthase* terminator; RB, right border

*OsERF94* contains a single exon, and three guide sequences were designed to target the exon for CRISPR/Cas9 gene editing (Fig. 2A). The CRISPR-P v2.0 (Liu et al. 2017) and the CRISPR RGEN tools (Park et al. 2015) were used for guide sequence design. The two guide sequences of sgRNA1 and sgRNA2 target the same position on the target gene, but the first nucleotide (5′ end) of the guide sequence of sgRNA2 was changed to adenine instead of cytosine (as in sgRNA1 and the WT). The first nucleotide of the guide sequence of sgRNA3 was also changed to adenine. Each guide sequence was introduced into the pRGEB31 vector (Addgene #51295) and driven by the *OsU3* promoter (Fig. 2B). The expression levels of *Cas9* and *Hpt* were driven by separate 35 S promoters.

A total of 81 transgenic plants were obtained through *Agrobacterium*-mediated transformation using mature seeds of ‘Nipponbare’. To identify mutations in *OsERF94*, PCR was performed using *OsERF94*-specific primers (Table S1) on T_0_ plants, followed by Sanger sequencing of the PCR products. The mutation types of T_0_ plants were determined by manually analyzing the Sanger sequencing results, which were then double-checked using the ICE tool (Roginsky 2018). The mutation efficiency of sgRNA1 was 25.64% (10 out of 39), whereas sgRNA2 exhibited a mutation efficiency rate of 72.73% (16 out of 22) (Table 1). These results suggest that the *U3* promoter prefers adenine as the transcription initiation site. The mutation efficiency of sgRNA3 was 35.00% (7 out of 20). Heterozygous mutations (54.55%) were the most frequently found mutation type, with 18 heterozygous mutants identified among the 33 T_0_ mutant lines (Table 2). Homozygous mutations were identified in five out of the 33 T_0_ mutant lines. Among them, four lines harbored homozygous mutations induced by sgRNA2, while one line carried a homozygous mutation induced by sgRNA3.

**Table 1:** Mutation efficiency rates of guide sequences

	Mutation efficiency (%)^z^
sgRNA1	10/39 (25.64)
sgRNA2	16/22 (72.73)
sgRNA3	7/20 (35.00)

^z^ The mutation efficiency of each guide sequence was calculated by dividing the number of gene-edited plants by the number of transgenic plants

**Table 2:** Analysis of mutation types in T_0_ mutant plants

	Homozygous		Heterozygous		Bi-allelic		Non-determined^z^		Total	
	No. of plants	Rate (%)	No. of plants	Rate (%)	No. of plants	Rate (%)	No. of plants	Rate (%)	No. of plants	Rate (%)
sgRNA1	0	0.00	8	80.00	2	20.00	0	0.00	10	
sgRNA2	4	25.00	5	31.25	5	31.25	2	12.50	16	
sgRNA3	1	14.29	5	71.43	0	0.00	1	14.29	7	
Total	5	15.15	18	54.55	7	21.21	3	9.09	33	100.00

^z^ Multi-alleic, chimera

**Fig. 3:**
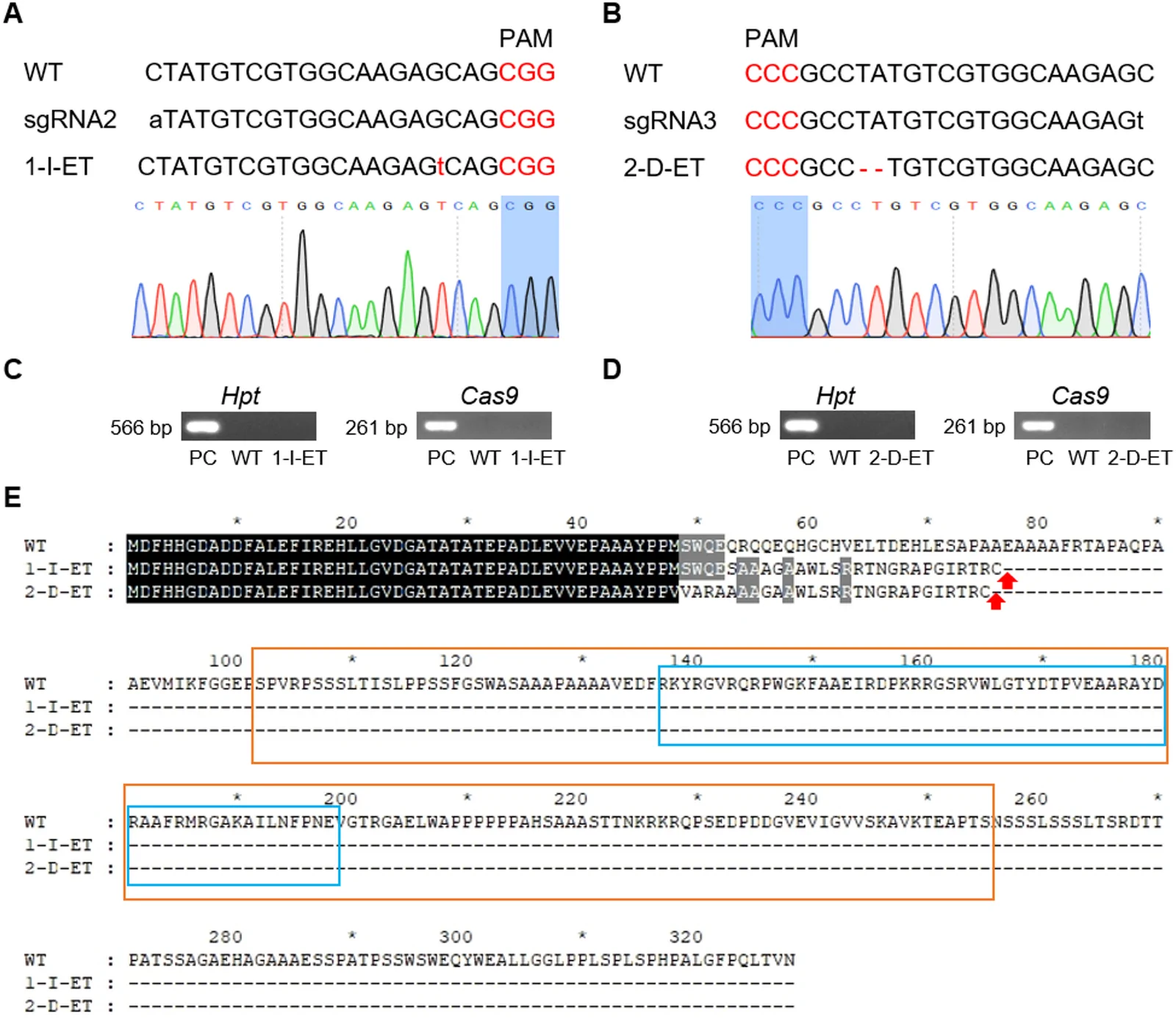
Mutations in *OsERF94* induced by CRISPR/Cas9. **A** A 1 bp insertion of thymine, induced by sgRNA2, was identified within *OsERF94* in 1-I-ET by Sanger sequencing. PAM sequences are indicated in red. **B** A 2 bp deletion (TA), induced by sgRNA3, was identified in 2-D-ET. T_1_ transgene-free mutants were selected using PCR screening with *Hpt*-specific and *Cas9*-specific primers in 1-I-ET (**C**) and 2-D-ET (**D**), respectively. Amino acid sequences of the WT, 1-I-ET, and 2-D-ET were aligned (**E**). The 1 bp insertion in 1-I-ET and the 2 bp deletion in 2-D-ET resulted in early termination, as indicated by the red arrows. An ethylene-responsive transcription factor and an AP2/ERF domain superfamily were predicted by InterProScan in *OsERF94*. The ethylene-responsive transcription factor is highlighted in the orange box, and the AP2/ERF domain superfamily is highlighted in the blue box. *Hpt*, *hygromycin phosphotransferase*; PC, positive control

A T_0_ heterozygous mutant line (#1-g2-C4) induced by sgRNA2 and a T_0_ homozygous mutant line (#1-g3-C13) were advanced to the next generation. *Cas9*-specific and *Hpt*-specific primers (Table S1) were used to identify transgene-free lines of T_1_ #1-g2-C4 and T_1_ #1-g3-C13, as the *Cas9* gene is located near the right border, while the *Hpt* gene is located near the left border in the pRGEB31 vector (Fig. 2B). PCR-negative lines were selected as transgene-free lines. To identify homozygous mutant lines among the transgene-free lines, Sanger sequencing was conducted. Transgene-free lines of T_1_ #1-g2-C4 with a homozygous 1 bp insertion were designated as 1-I-ET, while transgene-free lines of T_1_ #1-g3-C13 with a homozygous 2 bp deletion were designated as 2-D-ET (Fig. 3A and D). The 1 bp insertion in 1-I-ET and the 2 bp deletion in 2-D-ET caused frameshift mutations in *OsERF94*, leading to early termination (Fig. 3E). The ethylene-responsive transcription factor domain is detected between the 102nd and 255th amino acids, and the AP2/ERF domain superfamily is detected between the 137th and 198th amino acids, as determined by InterProScan (Quevillon et al. 2005). Both the ethylene-responsive transcription factor and the AP2/ERF domain superfamily were removed from the prematurely terminated proteins of 1-I-ET and 2-D-ET, respectively. These results indicate the loss-of-function mutations of *OsERF94*.

### Loss-of-Function of *OsERF94* Enhances Pre-Harvest Sprouting

**Fig. 4:**
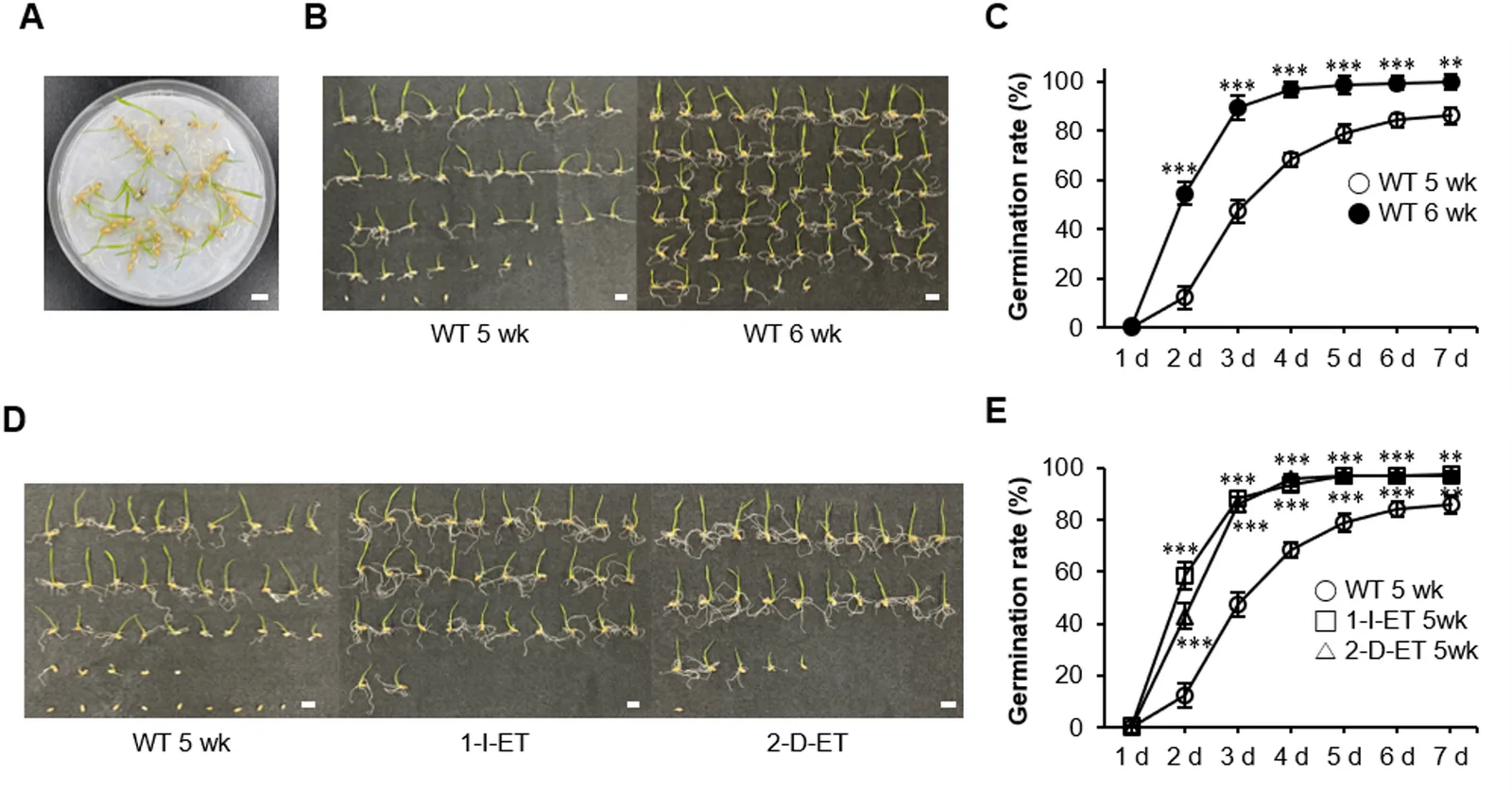
Increased pre-harvest sprouting in *OsERF94* gene-edited lines. Ten and nine panicles were collected from ‘Nipponbare’ at five and six weeks after heading, respectively. Seeds of the WT were germinated for seven days, and pictures were taken (**A**, **B**). The germination rate of the WT was evaluated daily for seven days (**C**). Ten and eleven panicles were collected from WT, 1-I-ET, and 2-D-ET at five weeks after heading, respectively. Seeds of the WT and gene-edited lines were germinated for seven days, and pictures were taken (**D**). The germination rate of the WT and gene-edited lines was evaluated daily for seven days (**E**). Error bars represent the standard error. Asterisks indicate significant differences compared to panicles collected from ‘Nipponbare’ at five weeks, as determined by Student’s *t*-test (* *p* < 0.05, ** *p* < 0.01, and *** *p* < 0.001)

To examine the PHS response based on the sampling timing, panicles were collected from ‘Nipponbare’ plants at five and six weeks after heading and were then subjected to germination experiments (Fig. 4A and B). When panicles were collected at six weeks after heading, the germination rate was 89.43% at three days after the PHS treatment and exceeded 95% starting from four days after the PHS treatment (Fig. 4C). The germination rates were significantly decreased at all time points except one day after PHS in WT panicles collected at five weeks after heading compared to those collected at six weeks. When panicles were collected at five weeks, the germination rate was 85.96% at seven days after the PHS treatment. Because seeds collected at six weeks after heading exhibited nearly complete germination, making it difficult to detect genotype-dependent differences, panicles collected at five weeks after heading were used for subsequent PHS assays. To ascertain the involvement of *OsERF94* in PHS in rice, *OsERF94* gene-edited lines were used for the PHS treatment. Panicles were collected at five weeks after heading from 1-I-ET (ten panicles) and 2-D-ET (eleven panicles). The germination rates were significantly increased at all time points, except for that at one day after PHS in 1-I-ET and 2-D-ET compared to WT panicles collected at five weeks (Fig. 4E). The germination rates of 1-I-ET and 2-D-ET at three days after the PHS treatment were 88.42% and 85.88%, respectively, similar to that (85.96%) of the WT at seven days. These results suggest that the loss-of-function of *OsERF94* increases susceptibility to PHS. On the other hand, no drastic phenotypic changes, such as severe dwarfism, were observed in either gene-edited line compared to the WT (Fig. S1). Although no severe morphological abnormalities were observed, plant height was slightly but significantly reduced in both mutant lines compared with the WT, whereas other agronomic traits, including tiller number, days to heading, and grains per panicle, showed no significant differences from the WT.

### Off-Target Analysis in *OsERF94* Gene-Edited Lines

We investigated whether the off-target effects of sgRNA2 and sgRNA3 resulted in mutations at off-target candidate sites in T_1_ gene-edited lines. Off-target candidate sites of sgRNA2 and sgRNA3 (Table S2) were predicted using CRISPR-GE (Xie et al. 2017). Whole-genome re-sequencing was conducted on 1-I-ET (#1-g2-C4-31, sgRNA2), 2-D-ET (#1-g3-C13-4, sgRNA3), and the WT, and the reads were mapped to the ‘Nipponbare’ reference genome. Few or no mutations were identified in any of the off-target candidate sites in both gene-edited lines compared to the WT. In most of the mapped reads, the mapped sequences were identical to those of the reference genome, and only a very small number of reads contained nucleotide substitutions rather than deletions or insertions. These nucleotide substitutions were considered to be sequencing errors. The mapping results of both gene-edited lines for off-target candidates with an off-score greater than 0.1 are presented in Fig. S2. In this study, *OsERF94* gene-edited lines were generated and validated to lack detectable off-target mutations.

### Transcriptome Analysis of *OsERF94*

**Fig. 5:**
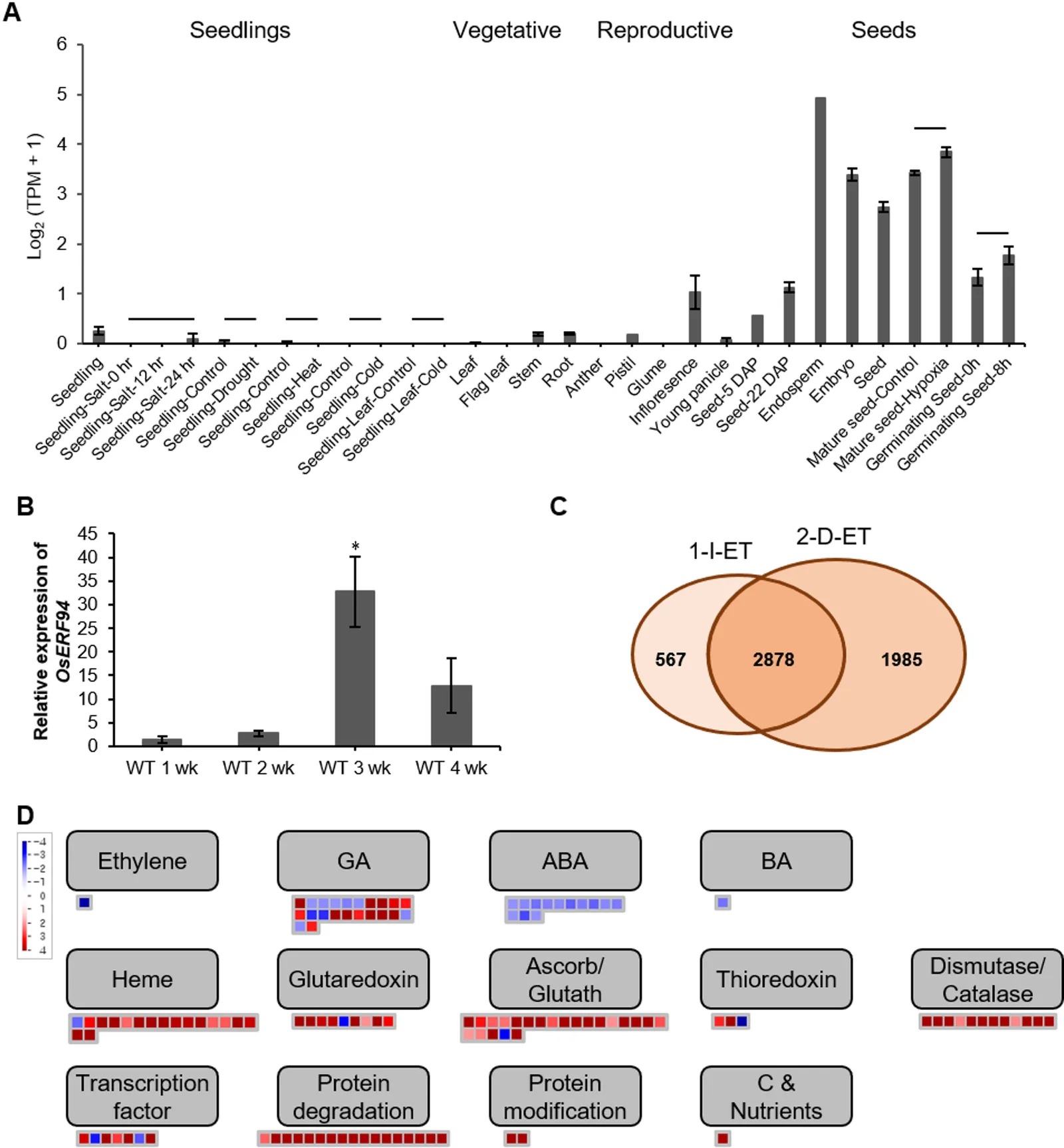
Expression analysis of *OsERF94* and transcriptomic profiling of *OsERF94* mutants. **A** Gene expression levels of *OsERF94* are presented as log_2_(TPM + 1), from 80 RNA sequencing libraries available at the Rice Genome Annotation Project (https://rice.uga.edu/index.shtml). Bars indicate average values, and error bars represent standard deviations. **B** The relative expression levels of *OsERF94* during grain development were quantified via qRT-PCR. The *OsActin* gene served as an internal control. Error bars represent the standard error of the mean of three biological replicates. An asterisk indicates a significant difference compared to samples collected one week after heading, as determined by the Student’s *t*-test (*p* < 0.05). **C** Venn diagram of DEGs identified from RNA sequencing of seeds collected three weeks after heading from 1-I-ET, 2-D-ET, and the WT. DEGs were defined by a comparison between each gene-edited line and the WT. **D** MapMan visualization of DEGs commonly identified in both gene-edited lines, categorized under ‘Regulation overview’. Red boxes indicate up-regulated DEGs, and blue boxes indicate down-regulated DEGs. The scale bar represents fold change values

The expression patterns of *OsERF94* across different tissues and developmental stages were examined using 80 rice RNA sequencing libraries available from the Rice Genome Annotation Project (RGAP, https://rice.uga.edu/index.shtml) (Hamilton et al. 2025). According to RGAP DB, *OsERF94* was more highly expressed during seed development than during seedling, vegetative, or reproductive stages (Fig. 5A). *OsERF94* expression is particularly high in the endosperm and embryo and is also elevated in mature seeds under hypoxic conditions. Specifically, the embryo RNA sequencing data (SRR9002107 and SRR9002108) were obtained from ‘Nipponbare’ embryos collected at 15 days after pollination, consistent with the cultivar used in this study.

To investigate the expression pattern of *OsERF94* further during grain development, RNA samples were collected at weekly intervals for four consecutive weeks starting one week after heading. *OsERF94* was highly expressed at three weeks after heading, and the expression level was significantly higher than that at one week after heading (Fig. 5B). In contrast, *OsERF94* was barely expressed at one and two weeks after heading, with CT values of qRT-PCR greater than 35. Therefore, we performed RNA sequencing using samples collected at three weeks after heading, when the expression level of *OsERF94* was the highest.

Using seeds collected three weeks after heading, RNA sequencing was conducted to investigate the role of *OsERF94* in PHS. Differentially expressed genes (DEGs) were identified by comparing each gene-edited line with the WT, using a threshold of |fold change| ≥ 2 and false discovery rate (FDR) *p*-value ≤ 0.05. A total of 3445 DEGs were identified in 1-I-ET, consisting of 2733 up-regulated and 712 down-regulated genes (Fig. 5C). In 2-D-ET, 4863 DEGs were identified, with 3413 up-regulated and 1450 down-regulated genes. The number of DEGs commonly identified in both 1-I-ET and 2-D-ET was 2878. To characterize the biological relevance, a MapMan analysis was conducted using the common DEGs (Fig. 5D). The average fold change values of the common DEGs between 1-I-ET and 2-D-ET were used for the MapMan analysis (Thimm et al. 2004) and are presented as red (up-regulated) or blue (down-regulated) in the corresponding MapMan diagram. Within the ‘Regulation overview’ category, 21 DEGs were identified in the ‘GA’ subcategory, representing the largest number among the subcategories related to plant hormones. In the ‘ABA’ subcategory, 13 DEGs were identified, all of which were down-regulated. The majority of these DEGs were related to ribosomal subunits (Table S3). In addition, a number of common DEGs were identified in redox-related subcategories, such as ‘Heme’, ‘Glutaredoxin’, and ‘Dismutase/Catalase’. In the ‘Dismutase/Catalase’ subcategory, most of the DEGs were associated with the lignin biosynthesis pathway (Table S3).

**Fig. 6:**
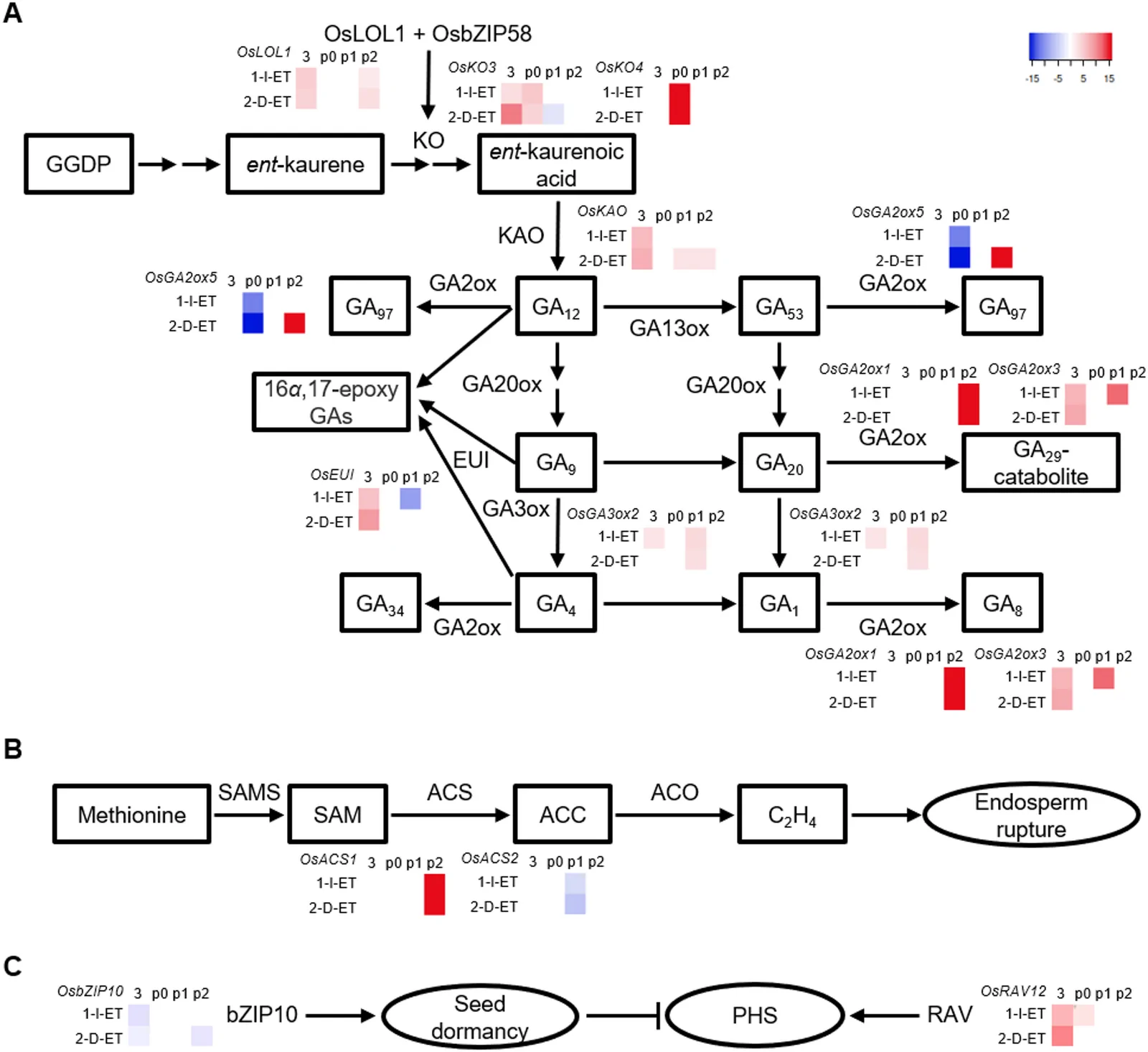
Gibberellin (GA) and ethylene biosynthesis pathway and differential gene expression in *OsERF94* gene-edited lines. **A** GA biosynthesis begins with *trans*-geranylgeranyl diphosphate (GGPP), which is converted to *ent*-kaurene and further processed into the active forms GA_1_ and GA_4_ by GA20ox and GA3ox. **B** Ethylene biosynthesis is initiated by the conversion of methionine into *S*-adenosyl-methionine (SAM) via the enzyme *S*-adenosyl-methionine synthetase (SAMS). SAM is then transformed into the ethylene precursor 1-aminocyclopropane-1-carboxylic acid (ACC) through the catalytic activity of ACC synthase (ACS). Subsequently, ACC is oxidized to ethylene by the action of ACC oxidase (ACO). **C** Expression of PHS-related genes (*OsbZIP10* and *OsRAV12*). DEGs simultaneously identified in the gene-edited plants are shown in italics, and the fold changes of the DEGs were visualized as a heatmap with the following color scale: red for up-regulation and blue for down-regulation. 3 indicates samples collected three weeks after heading, while P0, P1, and P2 indicate samples collected at zero, one, and two days after the PHS treatment using panicles collected five weeks after heading

Several genes involved in GA biosynthesis and deactivation were identified among the common DEGs between 1-I-ET and 2-D-ET (Fig. 6A). *OsLOL1* (*AGIS_Os08g005160*), *OsKO3* (*AGIS_Os06g033220*), and *OsKAO* (*AGIS_Os06g000950*) are involved in GA biosynthesis, while *OsEUI* (*AGIS_Os05g035360*) and *OsGA2ox3* (*AGIS_Os01g047760*) are involved in GA deactivation. All of the DEGs were up-regulated in both 1-I-ET and 2-D-ET. These results indicate that the loss of *OsERF94* is associated with altered expression of GA-related genes during developing seeds.

Moreover, additional RNA sequencing was conducted on seeds collected at zero, one, and two days after PHS using panicles harvested five weeks after heading from WT, 1-I-ET, and 2-D-ET. Before PHS, the GA biosynthesis genes *OsKO3* and *OsKO4* (*AGIS_Os06g033150*) were up-regulated in both 1-I-ET and 2-D-ET, while the GA deactivation gene *OsGA2ox5* (*AGIS_Os07g000350*) was down-regulated (Fig. 6A). One day after PHS, the GA activation gene *OsGA3ox2* (*AGIS_Os01g006890*) was up-regulated in both 1-I-ET and 2-D-ET (Fig. 6A). Two days after PHS, *OsLOL1*, involved in GA biosynthesis, and *OsGA2ox1* (*AGIS_Os05g005570*), involved in GA deactivation, were both up-regulated in 1-I-ET and 2-D-ET (Fig. 6A). These findings further indicate that such transcriptional changes are dynamic and stage-dependent during the PHS response.

In addition to GA-related genes, several genes involved in ethylene biosynthesis were differentially expressed in both 1-I-ET and 2-D-ET (Fig. 6B). *OsACS1* (*AGIS_Os03g044810*) was highly up-regulated in both lines, suggesting that *OsERF94* may also be associated with transcriptional changes in ethylene biosynthesis during the PHS response. Interestingly, several genes previously implicated in PHS were detected in the RNA sequencing results (Fig. 6C). *OsbZIP10* (*AGIS_Os01g056180*), also known as *OsABI5*, promotes seed dormancy and suppresses seed germination (Zinsmeister et al. 2016). In both gene-edited lines, *OsbZIP10* expression was down-regulated at three weeks after heading. Furthermore, *RAV* family genes have been reported to decrease PHS resistance (Tai et al. 2021; Li et al. 2025). Consistently, *OsRAV12* was up-regulated in both lines at three weeks after heading, which may contribute to the enhanced PHS phenotype in gene-edited lines.

To validate the RNA sequencing results, DEGs were analyzed by qRT-PCR (Fig. S3). *OsLOL1*, *OsKO3*, *OsKAO*, *OsGA2ox3*, *OsEUI*, *OsRAV12*, and *OsbZIP10* identified from the 3-week RNA sequencing samples (Fig. 6), as well as *OsKO3*, *OsGA3ox2*, and *OsKAO* from the P0, P1, and P2 RNA sequencing samples (Fig. 6), were subjected to qRT-PCR validation. The qRT-PCR results showed expression patterns largely consistent with the RNA sequencing analysis.

### Transient Luciferase Reporter Assay for GA- and Ethylene-Related Gene Promoters

**Fig. 7:**
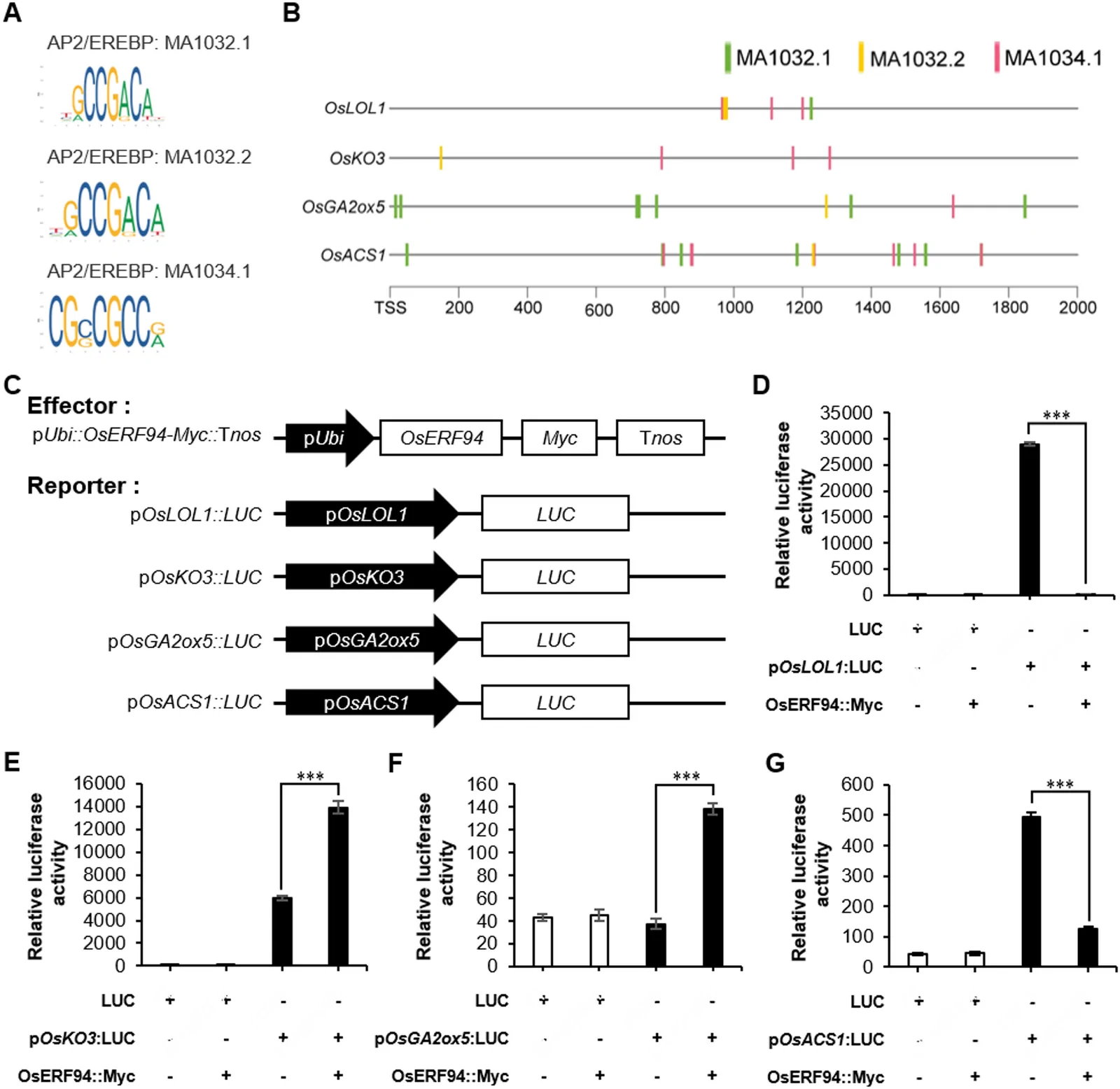
Transcriptional activation activity of OsERF94. **A** Matrix profiles of three AP2/EREBP transcription factor binding motifs retrieved from the JASPAR database for *japonica* rice. **B** Distribution of predicted AP2/EREBP binding sites in the 2-kb upstream promoter regions of selected candidate genes involved in gibberellin- and ethylene-related pathways, including *OsLOL1*, *OsKO3*, *OsGA2ox5*, and *OsACS1*. Colored vertical bars indicate the positions of motif matches for MA1032.1 (green), MA1032.2 (yellow), and MA1034.1 (pink), relative to the transcription start site (TSS). **C** Schematic representation of the effector and reporter constructs used in the luciferase reporter assay. Relative luciferase activities were measured using a single-luciferase system in rice protoplasts after co-transfection with effector and reporter constructs. The promoters of *OsLOL1* (**D**), *OsKO3* **(E**), *OsGA2ox5* (**F**), and *OsACS1* (**G**) were used to generate reporter constructs. Data represent the mean ± SEM. Statistical significance was determined using Student’s *t*-test (* *p* < 0.05, ** *p* < 0.01, and *** *p* < 0.001)

As OsERF94 is an AP2/ERF transcription factor and our RNA sequencing showed coordinated changes in GA biosynthesis and GA deactivation genes, together with ethylene nodes, we scanned the − 2 kb promoter regions of these genes (*OsLOL1*, *OsKO3*,* OsGA2ox5*, and *OsACS1*) for AP2/EREBP binding motifs. Using the JASPAR *japonica* matrices MA1032.1, MA1032.2, and MA1034.1 (Fig. 7A), putative AP2/EREBP binding motifs were identified in all promoters examined (Fig. 7B), suggesting a potential cis-regulatory basis for the coordinated expression observed in the *OsERF94* mutant lines.

To further investigate whether OsERF94 may be associated with changes in the promoter activity of GA- and ethylene-related genes identified in the RNA sequencing analysis, transient luciferase reporter assays using a single-luciferase system were performed in rice protoplasts using promoter constructs derived from *OsLOL1*, *OsKO3*, *OsGA2ox5*, and *OsACS1* (Fig. 7C). Co-expression of *OsERF94* with the *OsLOL1* promoter resulted in a significant reduction in luciferase activity compared with the reporter construct alone (Fig. 7D). In contrast, luciferase activity driven by the *OsKO3* promoter significantly increased when *OsERF94* was co-expressed (Fig. 7E). For the *OsGA2ox5* promoter, luciferase activity also increased following *OsERF94* co-expression, although the basal activity of this promoter was close to background levels (Fig. 7F). Luciferase activity driven by the *OsACS1* promoter significantly decreased in the presence of *OsERF94* (Fig. 7G). These results suggest that OsERF94 may be associated with changes in the promoter activities of several GA- and ethylene-related genes. However, because the single-luciferase system was performed without REN normalization, transient transformation efficiency may have affected luciferase activity. In addition, direct binding of OsERF94 to these promoters was not tested in this study.

### Endogenous GAs and ABA Quantification in Seeds Subjected to PHS

**Fig. 8:**
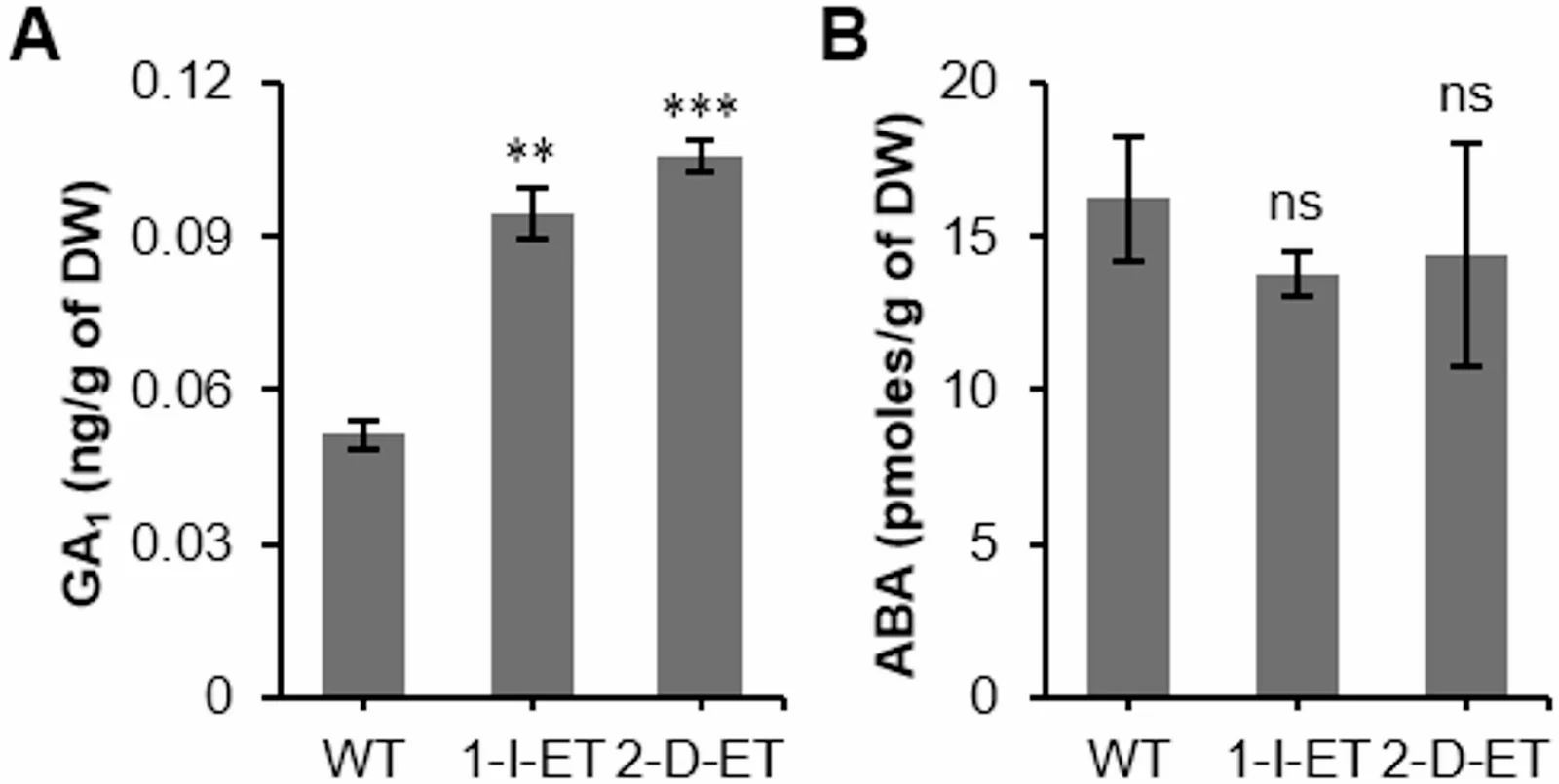
Bioactive gibberellic acid (GA) and abscisic acid (ABA) quantification in pre-harvest sprouting (PHS) samples. Seeds were collected two days after PHS and freeze-dried. Bioactive GA_1_ (**A**) and ABA (**B**) concentrations were evaluated using three biological replicates. Data represent the mean ± SEM. Statistical significance was determined using Student’s *t*-test (* *p* < 0.05, ** *p* < 0.01, and *** *p* < 0.001). ns indicates not significant

To quantify endogenous GAs and ABA during PHS, panicles collected at five weeks after heading were subjected to the PHS treatment. At two days after PHS treatment, five endogenous GAs (Table S4) and ABA (Fig. 8B) were measured. Among the quantified GAs, the level of bioactive GA_1_ was significantly higher in the *OsERF94* mutant lines than in the WT (Fig. 8A). In contrast, ABA levels were slightly lower in the mutant lines (13.78 ± 0.70 pmol g⁻¹ DW in 1-I-ET and 14.41 ± 3.63 pmol g⁻¹ DW in 2-D-ET) compared to WT (16.25 ± 2.02 pmol g⁻¹ DW), although these differences were not statistically significant (Fig. 8B). These findings suggest that the loss of *OsERF94* is associated with increased levels of bioactive GA_1_, which may contribute to the enhanced PHS susceptibility in the *OsERF94* mutant lines.

### Germination Response of the *OsERF94* Mutants to Exogenous GA_3_

To determine whether *OsERF94* mutants exhibit altered sensitivity to gibberellin, germination assays were performed on half-strength MS medium supplemented with GA₃ (0, 1, and 5 µM). Both WT and mutant lines exhibited comparable germination responses across all GA₃ concentrations, with no significant differences observed between genotypes (Fig. S4). These results indicate that the enhanced PHS phenotype observed in the *OsERF94* mutants is unlikely to be due to altered sensitivity to exogenous GA₃. Rather, it may be associated with changes in endogenous GA metabolism, as suggested by the transcriptome and hormone quantification analyses.

## Discussion

### CRISPR/Cas9-Mediated Mutagenesis of *OsERF94* Enhances Susceptibility to Pre-Harvest Sprouting

CRISPR/Cas9 gene editing has been widely utilized in plant molecular research, as CRISPR/Cas9 enables precise and accurate modifications of target genes (Bortesi et al. 2016; Romero and Gatica-Arias 2019). We successfully induced mutations in *OsERF94* via CRISPR/Cas9-mediated mutagenesis. In T_0_ plants, the mutation efficiency rates across all three sgRNAs was 40.74%, with the mutation efficiency rates of sgRNA1 and sgRNA2 being 25.64% and 72.73%, respectively (Table 1). Only one nucleotide differed between the guide sequences of sgRNA1 and sgRNA2. While the guide sequence of sgRNA1 is a perfect match to *OsERF94*, that of sgRNA2 has a single mismatch, where the cytosine at the 5′ end is substituted with adenine. The mutation efficiency of sgRNA2 was higher than that of sgRNA1 by 47.09%. It is known that the *U3* promoter in the CRISPR/Cas9 vector initiates transcription at a defined adenine nucleotide (Belhaj et al. 2013). The guide sequence of sgRNA2 begins with adenine, which may lead to higher expression of sgRNA2 compared to sgRNA1, thereby resulting in higher mutation efficiency.

The loss-of-function of *OsERF94* was associated with increased susceptibility to PHS. Our results are consistent with our previous GWAS but appear to be inconsistent with the haplotype analysis of PHS (Fig. 1). It seems that the non-synonymous mutation in haplotype 2 enhances PHS resistance in rice, similar to the PHS-resistant allele of *OsERF1*, which carries a non-synonymous mutation and a 6 bp insertion (Kim et al. 2024). The CRISPR-Cas9/HDR and geminiviral replicon system was applied to ‘Dongjin’, which does not contain either the non-synonymous mutation or the 6 bp insertion, resulting in the ERF1-hdr line carrying both mutations. PHS resistance was enhanced in the ERF1-hdr line compared to ‘Dongjin’. In the present study, CRISPR/Cas9 gene editing of *OsERF94* induced early termination, which is expected to abolish the *OsERF94* function entirely through complete gene knockout. As shown in Fig. 1, if the gene-edited lines had the haplotype 2 variant, they may exhibit enhanced resistance to PHS. However, because our lines represent a complete knockout, it is possible that they are even more susceptible to PHS than the haplotype 1 lines, which are already known to be PHS-susceptible.

### Transcriptional Changes During PHS Associated with *OsERF94* Loss-of-Function

*OsERF94* appears to be associated with both the biosynthesis and deactivation of GAs during seed development. At three weeks after heading, the GA biosynthesis-related genes *OsLOL1*, *OsKO3*, and *OsKAO*, as well as the GA deactivation-related gene *OsEUI*, were all up-regulated in both 1-I-ET and 2-D-ET (Fig. 6A). OsLOL1, a C2C2-type finger protein, participates in GA biosynthesis, influencing seed germination in rice (Wu et al. 2014). OsLOL1 interacts with OsbZIP58 to activate OsKO2 (Wu et al. 2014). A homolog of *OsKO2*, *OsKO3*, also functions in the GA biosynthetic pathway (Chen et al. 2019), and CRISPR/Cas9-mediated knockout of *OsKO3* resulted in delayed germination. *OsKAO*, which catalyzes the formation of GA_12_, is a key GA biosynthetic enzyme in rice (Sakamoto et al. 2004). Unlike other GA-metabolic enzymes encoded by multigene families, *OsKAO* is represented by a single gene in rice. Mutation of *OsKAO* resulted in a severely dwarfed phenotype accompanied by substantial reductions in bioactive GA levels (Sakamoto et al. 2004). Together, *OsLOL1*, *OsKO3*, and *OsKAO* play important roles in GA biosynthesis, and the up-regulation of these genes in both gene-edited lines may contribute to reduced dormancy by influencing GA-related gene expression patterns. In contrast, OsEUI functions as a GA deactivating enzyme and reduces the activity of bioactive GA_4_ in rice (Zhu et al. 2006). As O*sEUI* has been reported to suppress GA accumulation, its up-regulation in the gene-edited lines may reflect compensatory regulation within GA metabolic pathways. These findings suggest that *OsERF94* is associated with transcriptional changes in GA biosynthetic and catabolic genes in developing seeds, potentially influencing the balance between seed dormancy and the germination potential.

*OsERF94* loss-of-function was associated with changes in GA-related gene expression at zero, one, and two days after PHS using panicles harvested five weeks after heading. At five weeks after heading (before PHS), *OsKO3* and its homolog *OsKO4* were up-regulated, while the GA deactivation gene *OsGA2ox5* was down-regulated (Fig. 6A). *OsGA2ox5* acts on GA_12_ and GA_53_ to produce GA_110_ and GA_97_, respectively (Dong and Zeevaart 2005; Shan et al. 2014). Moreover, *OsGA3ox2*, which is involved in the production of bioactive GAs (GA_1_ and GA_4_), was up-regulated one day after PHS (Fig. 6A) (Itoh et al. 2001). The up-regulation of GA biosynthesis genes *OsKO3*, *OsKO4*, and *OsGA3ox2*, along with the down-regulation of the GA catabolic gene *OsGA2ox5*, is consistent with a shift toward increased GA biosynthetic potential in seeds. Germination rates were sharply and significantly increased from two days after PHS in both gene-edited lines compared to those of the WT (Fig. 4E). Taken together, the loss-of-function of *OsERF94* is associated with transcriptional changes in GA biosynthetic and catabolic genes, which may contribute to enhanced PHS susceptibility. At two days after PHS, the GA deactivation gene *OsGA2ox1* became up-regulated. This transition from down-regulation (*OsGA2ox5* before PHS) to subsequent up-regulation of GA deactivation genes may reflect feedback-like regulatory adjustments within GA metabolic pathways, potentially contributing to the maintenance of GA homeostasis.

The differential expression levels of ethylene biosynthesis genes in both *OsERF94* gene-edited lines under PHS conditions appear to contribute to seed germination. Endosperm rupture during seed germination stems from embryo growth and the weakening of the endosperm cap (Zhang et al. 2014). In *Sisymbrium officinale*, *SoACS7* was not expressed before endosperm rupture but was strongly expressed when rupture reached 50–100% (Iglesias-Fernández and Matilla 2010). In our study, seed germination was defined as the point at which the coleoptile length exceeded 2 mm. One day after PHS, the germination rates of 1-I-ET, 2-D-ET, and the WT were 0.32%, 0%, and 0%, respectively (Fig. 4E). Two days after PHS, the germination rates increased to 58.44% in 1-I-ET, 42.79% in 2-D-ET, and 12.46% in the WT. These results suggest that endosperm rupture occurred more frequently in the gene-edited lines than in the WT on the second day after PHS. Moreover, *OsACS1* was strongly up-regulated in both gene-edited lines at two days after PHS (Fig. 6B).

Notably, *OsbZIP10* (*OsABI5*), a positive regulator of seed dormancy that increases resistance to PHS, was down-regulated in both mutants (Fig. 6C) (Zinsmeister et al. 2016; Du et al. 2018). Mutation induced by *Tnt1* insertion in *ABI5* of *Medicago truncatula* led to impaired dormancy acquisition, highlighting the essential role of *ABI5* in seed maturation (Zinsmeister et al. 2016). The rice *phs8* mutant exhibits PHS phenotype, whereas overexpression of *OsABI5* in the mutant background significantly decreased PHS rate (Du et al. 2018). Overexpression of *OsABI5* in ‘Nipponbare’ significantly reduced germination compared with the WT (Li et al. 2022). These studies indicate that *OsbZIP10* functions as a negative regulator of PHS. Therefore, the reduced expression of *OsbZIP10* in the *OsERF94* gene-edited lines may explain the increased PHS rate. Conversely, *osrav9* mutant lines induced by CRISPR/Cas9 exhibited enhanced PHS resistance and reduced seed germination compared to the WT (Li et al. 2025), indicating that *RAV* genes may positively regulate germination. In our study, *OsRAV12* was up-regulated in both gene-edited lines, which may contribute to the enhanced PHS susceptibility in gene-edited lines.

The gene expression patterns of GA biosynthesis genes (*SoGA3ox2*, *SoGA20ox2*, and *SoGA2ox6*) and ethylene biosynthesis genes (*SoACO2* and *SoACS7*) were analyzed during seed germination in *Sisymbrium officinale* (Iglesias-Fernández and Matilla 2010). Seed samples were collected at 0, 6, 12, 18, 20, 22, and 26 h of germination. Endosperm rupture was observed at 20 h, reached approximately 50% at 22 h, and was nearly complete by 26 h. *SoGA3ox2* was strongly expressed at six hours of imbibition, but its expression declined afterward, maintaining about half the level from 12 to 26 h. In contrast, *SoGA2ox6* transcripts were barely detectable at six hours but peaked at 20 and 26 h. *SoACS7* was not expressed before endosperm rupture but showed strong expression at 22 and 26 h. In our study, *OsGA3ox2* was up-regulated one day after PHS in both *OsERF94* gene-edited lines, while *OsGA2ox1* and *OsACS1* were strongly up-regulated two days after PHS. This sequential up-regulation pattern of *OsGA3ox2*, *OsGA2ox1*, and *OsACS1* in *OsERF94* gene-edited lines, together with the observed germination rates under PHS conditions, closely resembles the sequential expression pattern of *SoGA3ox2*, *SoGA2ox6*, and *SoACS7* during seed germination in *Sisymbrium officinale*. These findings suggest that GA-associated responses may occur earlier than ethylene-related responses during seed germination, although the precise regulatory relationship between these hormonal pathways remains to be clarified.

In addition, transient luciferase reporter assays in rice protoplasts using a single-luciferase system suggested that OsERF94 may be associated with changes in the promoter activities of *OsLOL1* and *OsACS1*, while increasing the luciferase activity of the *OsGA2ox5* promoter. These patterns are broadly consistent with the RNA sequencing results obtained from seed development and PHS samples of the *OsERF94* mutant lines, showing increased expression of *OsLOL1* and *OsACS1* and decreased expression of *OsGA2ox5* in both *OsERF94* mutant lines. In contrast, the *OsKO3* promoter showed increased luciferase activity, whereas *OsKO3* expression was also elevated in the *OsERF94* mutant lines according to the RNA sequencing results (Fig. 6A). This discrepancy may reflect the complexity of transcriptional regulation in plants, where additional regulatory elements or interacting transcription factors may influence gene expression beyond the transient protoplast assay system. Because the luciferase assay was performed using a single-luciferase system, the observed reporter activities may have been partially affected by variation in transient transformation efficiency. Together, these findings suggest that *OsERF94* may influence the transcription of several genes associated with GA- and ethylene-related pathways during PHS, although the precise regulatory mechanisms remain to be clarified.

Moreover, the bioactive GA_1_ concentration at two days after PHS was significantly higher in the *OsERF94* mutant lines than in the WT (Fig. 8A). Notably, RNA sequencing analysis revealed that the GA biosynthetic gene *OsGA3ox2* was up-regulated in the mutant lines at one day after PHS treatment (Fig. 6A). Because endogenous GA_1_ levels were measured at two days after PHS, this earlier induction of *OsGA3ox2* may contribute to the subsequent increase in GA_1_ accumulation in the mutant lines. The elevated GA_1_ levels are consistent with the earlier induction of *OsGA3ox2* and may therefore contribute to the enhanced PHS susceptibility observed in the *OsERF94* mutant lines. Although the transcriptome, luciferase reporter, and hormone quantification data collectively support a role for *OsERF94* in GA- and ethylene-related pathways during PHS, direct binding of OsERF94 to the promoters of these genes was not demonstrated in this study. In addition, the present work focused primarily on early germination and PHS phenotypes, and broader agronomic consequences of *OsERF94* manipulation remain to be investigated. Further studies using DNA-binding assays and additional field-based phenotyping will help clarify the precise regulatory mechanism and the breeding value of *OsERF94*.

In conclusion, our study supports the idea that the loss-of-function of *OsERF94* increases susceptibility to PHS in rice and is associated with altered expression of GA biosynthetic and catabolic genes. *OsERF94* may influence GA-related processes during seed development and the early stages of germination. The altered expression levels of key GA-related genes, specifically *OsLOL1*, *OsKO3*, *OsGA3ox2*, and *OsGA2ox5*, suggest that *OsERF94* may contribute to the balance between seed dormancy and germination. To the best of our knowledge, little is known about the expression patterns and functional roles of *OsERF94* in rice or other plant species. Our findings contribute to a better understanding of the role of *OsERF94* in the regulation of PHS. In addition, two PHS-associated genes (*OsERF1* and *OsERF94*) were identified near the SNP marker Chr4_Pos27378200, providing valuable insights for rice-breeding programs aiming to improve PHS resistance.

## Materials and Methods

### Plant Materials and Agronomic Traits

Rice cultivar ‘Nipponbare’ (*Oryza sativa* L., spp. *japonica*) plants were grown in a growth chamber maintained under 12 h of light (29 ± 1 °C) and 12 h of darkness (23 ± 1 °C). Agronomic traits of WT and *OsERF94* mutant lines were evaluated. Plant height, tiller number, days to heading, grains per panicle, and thousand-grain weight were examined. For grains per panicle and thousand-grain weight, five plants from WT and each mutant line were used, and three panicles per plant were randomly selected for measurement.

### Pre-Harvest Sprouting Assay

Panicles were collected at 35 and 42 days after heading (DAH) from WT and at 35 DAH from mutant lines induced by CRISPR/Cas9 for PHS screening. The primary branch of the panicle was cut and placed on autoclaved Whatman filter paper No. 1 (Whatman, Little Chalfont, UK) in a petri dish (150 × 20 mm), after which an amount of 30 ml of tap water was added to the petri dish. The lid was covered to maintain humidity. The petri dishes, covered with the lids, were placed in a growth chamber maintained at 12 h of light (29 °C, ± 1) and 12 h of darkness (23 °C, ± 1). Germination was evaluated every day for seven days using plump seeds, excluding sterile seeds. Seeds were evaluated as germinated when the length of the coleoptile exceeded 2 mm.

### Exogenous GA_3_ Germination Assay

Dehusked mature seeds were surface-sterilized with 70% ethanol for 5 min and rinsed with distilled water three times, followed by sterilization with 10% commercial Chlorox for 5 min and subsequent rinsing with distilled water three times. The sterilized seeds were placed on half-strength Murashige and Skoog (MS) medium supplemented with 0, 1, and 5 µM GA_3_. Plates were incubated in a growth chamber at 25 °C. For each treatment, 40 seeds were used with three biological replicates per line (WT and both mutant lines). Seeds with shoot length ≥ 2 mm were recorded as germinated.

### CRISPR/Cas9 Vector Construction and Rice Transformation

Guide sequence candidates targeting *OsERF94* (*Os04g0547600*) were designed using the CRISPR-P v2.0 (Liu et al. 2017) and the CRISPR RGEN tools (Park et al. 2015). Two target sites within the exon of *OsERF94* (5′– CTATGTCGTGGCAAGAGCAG**CGG** − 3′ and 5′– **CCC**GCCTATGTCGTGGCAAGAGC − 3′, PAM regions are indicated in bold) were used to design the guide sequences. A total of three guide sequences (sgRNA1 : 5′– CTATGTCGTGGCAAGAGCAG − 3′; sgRNA2 : 5′– aTATGTCGTGGCAAGAGCAG − 3′; 5′– aCTCTTGCCACGACATAGGC − 3′) were designed for CRISPR/Cas9 vector construction using the pRGEB31 plasmid (addgene, #51295). The *OsU3* promoter drives the single guide RNA in the pRGEB31 vector. The first nucleotide of sgRNA2 and sgRNA3 was changed to A. sgRNA1 and sgRNA2 target the same position in the *OsERF94* exon. Each guide sequence was introduced into the pRGEB31 vector. The CRISPR/Cas9 vectors for *OsERF94* gene editing were transformed into the *Agrobacterium* strain EHA105 by a freeze–thaw method (Chen et al. 1994). *Agrobacterium*-mediated transformation using mature seeds of ‘Nipponbare’ was conducted, as described in earlier work (Lee et al. 1999). Transformed calli were selected on callus induction media containing 30 mg L^− 1^ of hygromycin during the first round of selection, and on media containing 50 mg L^− 1^ of hygromycin during the second round. Shoots were regenerated on shoot induction media containing 50 mg L^− 1^ of hygromycin at 28 ℃ under a light/dark cycle (12 h of day/12 hours of night).

### Identification of Gene-Edited Mutants

Genomic DNA was extracted from young leaves of putative transgenic plants using the HiGene™ Genomic DNA Prep kit (BIOFACT, Seoul, Republic of Korea) according to the manufacturer’s instructions. PCR was conducted to detect transgenic plants with *Cas9*-specific primers. To identify mutations in *OsERF94*, PCR was conducted with *OsERF94*-specific primers using T_0_ plants, followed by Sanger sequencing of the PCR products. Mutation types of T_0_ plants, such as heterozygous mutation and homozygous mutation, were distinguished by manually analyzing the Sanger sequencing results and were double-checked using the ICE (Inference of CRISPR Edits) tool (Roginsky 2018). The list of primers for PCR is available in Table S1. PCR was carried out with TaKaRa *Ex* Taq (Takara Bio, Otsu, Japan) under the following conditions: 95 °C for 5 min, followed by 30 cycles of 95 °C for 30 s, 56–62 °C for 30 s, and 72 °C for 1 min, with a final extension at 72 °C for 5 min.

To obtain transgene-free homozygous mutants, genomic DNA was extracted from young leaves of T_1_ plants, and PCR was performed with the *Cas9*-specific primers and *Hpt*-specific primers. The list of primers for PCR is available in Table S1. PCR-negative plants were selected and used for further PCR with the *OsERF94*-specific primers. Sanger sequencing results of the PCR products were analyzed manually and double-checked using the ICE tool.

### Potential Off-Targets and Whole-Genome Re-Sequencing

Potential off-targets of sgRNA2 and sgRNA3 were predicted using CRISPR-GE (Xie et al. 2017). Two transgene-free homozygous mutant lines, 1-I-ET-31 (#1-g2-C4-31) and 2-D-ET-4 (#1-g3-C13-4), and the WT were used for whole-genome re-sequencing to search for off-targets. Genomic DNA (100 ng) was fragmented and subsequently used for library preparation with the TruSeq Nano DNA Library Prep kit (Illumina, CA, USA) following the manufacturer’s protocol. The quality of libraries was examined via electrophoresis using the Agilent High Sensitivity DNA kit (Agilent, CA, USA). Library quantification was performed using the KAPA Library Quantification kit (Kapa Biosystems, MA, USA) according to the manufacturer’s protocol. Sequencing was conducted in paired-end mode (2 × 150 bp) on the Illumina NovaSeq X Plus platform (Illumina, CA, USA). The quality of the raw data was evaluated using FastQC. Low-quality reads were trimmed with Cutadapt using a Q20 cutoff and a minimum remaining length of 15 bp. The resulting clean reads were then aligned to the ‘Nipponbare’ reference genome (available at http://www.ricesuperpir.com/web/nip) (Shang et al. 2023) using BWA and were visualized with Geneious Prime v2023.0.4.

### RNA Extraction and Quantitative Real‑Time PCR

Hulled seeds were collected at 7, 14, 21, 28 DAH from ‘Nipponbare’ and were frozen in liquid nitrogen. Seeds from each sampling point were pooled prior to RNA extraction for transcriptome sequencing. The seeds were ground into a fine powder using a mortar and pestle. Total RNA was extracted using the Ribospin™ Seed/Fruit kit (Geneall, Seoul, Republic of Korea) following the manufacturer’s instructions. RNA was converted to cDNA by reverse transcription using oligo (dT) primers with the Power cDNA Synthesis Kit (iNtRON Biotechnology, Seoul, Republic of Korea). To evaluate the expression of the *OsERF94* gene during seed development, qRT-PCR was performed with the RealMOD™ Green W^2^ 2x qPCR mix (iNtRON Biotechnology, Seoul, Republic of Korea) on a Rotor-Gene Q machine (QIAGEN, Hilden, Germany). The qRT-PCR conditions were 95 ℃ for 10 min followed by 40 cycles of 95 ℃ for 20 s, 60 ℃ for 40 s, and 72 °C for 20 s, with a final extension at 72 °C for 5 min. Gene expression levels were normalized to *OsACT1* (*Os03g0718100*). The primer information is presented in Table S1. Total RNA was extracted from three panicles as biological replicates, and qRT-PCR was conducted with three technical replicates for each sample. Gene expression levels were quantified using the 2^−ΔΔCT^ method (Livak and Schmittgen 2001).

To validate the expression levels of DEGs identified by RNA sequencing, the RNA samples used for RNA sequencing were subjected to qRT-PCR as described above with minor modifications. Amplification was performed at 95 °C for 2 min, followed by 40 cycles of 95 °C for 10 s and 60 °C for 20 s. Primer information is provided in Table S1.

### RNA Sequencing

Seeds were collected at 21 DAH from 1-I-ET, 2-D-ET, and the WT, with three biological replicates for each line. Total RNA was isolated using the Ribospin™ Seed/Fruit kit (Geneall, Seoul, Republic of Korea) according to the manufacturer’s instructions. The total RNA concentration was measured using the Quant-iT RiboGreen RNA Assay kit (Invitrogen, MA, USA). RNA integrity was examined using the TapeStation RNA ScreenTape system (Agilent, CA, USA). High-quality RNA samples with an RIN greater than 7.0 were used to prepare libraries, with 0.5 µg of total RNA per sample, using the TruSeq Stranded Total RNA Library Prep Plant kit (Illumina, CA, USA). Libraries were quantified using the KAPA Library Quantification kit (Kapa Biosystems, MA, USA), and the quality of libraries was evaluated using the D1000 ScreenTape system (Agilent, CA, USA). Indexed libraries were then sequenced in paired-end mode (2 × 150 bp) on an Illumina HiSeq X Ten (Illumina, CA, USA). Adapter sequences and low-quality bases were removed using Trimmomatic v0.38 (Bolger et al. 2014). Cleaned reads were aligned to the *Oryza sativa* T2T-NIP reference genome using HISAT2 v2.1.0 (Shang et al. 2023). SAM files were sorted and indexed using SAMtools v1.9 (Li et al. 2009) and transcript assembly and quantification were performed using StringTie v2.1.3b (Pertea et al. 2015). The gene expression analysis was conducted using DESeq2 (Love et al. 2014). DEGs were identified based on the criteria of |fold change| ≥ 2 and FDR *p*-value < 0.05. For the MapMan analysis (Thimm et al. 2004), bincode mapping was conducted using the Mercator webtool (https://www.plabipd.de/portal/mercator-sequence-annotation) (Lohse et al. 2014). Common DEGs between 1-I-ET and 2-D-ET were selected, and common DEGs with an average fold change greater than 2 were subjected to a MapMan analysis to identify affected metabolic pathways.

Additionally, seeds were collected at zero, one, and two days after the PHS treatment using panicles harvested at five weeks after heading from 1-I-ET, 2-D-ET, and the WT, with two biological replicates for each line. Total RNA was extracted from each line and was used for RNA sequencing on the same platform described above. DEGs were identified using the criteria of |fold change| ≥ 2 and *p*-value < 0.05.

### Transient Luciferase Reporter Assay in Rice Protoplasts

To generate reporter constructs, the promoter regions of *OsLOL1*, *OsKO3*, *OsGA2ox5*, and *OsACS1* were amplified by PCR. Primer information is provided in Table S1. The amplified promoter fragments were fused to the luciferase reporter gene by cloning into the restriction enzyme–digested pGL4.23-Luc vector using the In-Fusion cloning method. Rice protoplasts were isolated from two-week-old leaf blades grown in the dark at 28 °C. Leaves were finely chopped and incubated in enzyme solution (1% cellulose RS, 1% macerozyme, 0.1% pectolyase Y-23, 0.4 M mannitol, 0.1% CaCl₂, 0.1% BSA, and 5 mM MES, pH 5.7). The samples were vacuum infiltrated for 30 min and subsequently incubated for 5 h at 28 °C in the dark. After washing with an equal volume of W5 solution (5 mM KCl, 154 mM NaCl, 125 mM CaCl_2_, and 2 mM MES, pH 5.7), harvested protoplasts were resuspended in MMG solution (0.4 M mannitol, 15 mM MgCl_2_, and 4 mM MES, pH 5.7) to obtain a density of 3 × 10^6^ cells mL^− 1^, as determined using a hemocytometer. For transient expression assays, protoplasts were co-transfected using a polyethylene glycol–calcium-mediated method (Choi et al. 2014). Transfected protoplasts were incubated overnight in WI incubation buffer (0.5 M mannitol, 20 mM KCl, and 4 mM MES, pH 5.7) and then harvested. The harvested protoplasts were resuspended in Cell Culture Lysis 1× Reagent (Promega, WI, USA) and used for luciferase assays. Luciferase activity was measured using a single-luciferase system with the Luciferase Assay Kit (Promega, WI, USA). The luminescence generated by luciferase was detected using a GloMax^®^-Multi+ detection system (Promega, WI, USA). All transient expression assays were repeated four times.

### Quantification of Endogenous GAs and ABA

Panicles collected at five weeks after heading were subjected to the PHS assay as described above. Seeds were collected two days after PHS treatment, dehusked, and freeze-dried. Extraction and quantification of endogenous GAs were conducted as previously described by Lee et al. (1998). Deuterated GA internal standards were added to the extracts, and endogenous GAs were subsequently quantified by gas chromatography–mass spectrometry. The endogenous ABA concentration was measured using a Phytodetek ABA Test Kit (Agdia Inc., IN, USA) according to the manufacturer’s instructions. Three biological replicates were included for each plant line.

## Supplementary Information

Below is the link to the electronic supplementary material.

## Data Availability

The datasets generated during this study are available from the corresponding author upon reasonable request.

## Abbreviations

ABA: Abscisic acid
AP2: APETALA2
DAH: Days after heading
DEG: Differentially expressed gene
ERF: Ethylene response factor
FDR: False discovery rate
GA: Gibberellin
GWAS: Genome-wide association study
PHS: Pre-harvest sprouting
RGAP: Rice genome annotation project
